# The Influence of nc-AlCrTiN/α-BN Coatings on Increasing the Durability of WC/Co Cutting Inserts in the Inconel Alloy Machining Process

**DOI:** 10.3390/ma17112587

**Published:** 2024-05-28

**Authors:** Joanna Kacprzyńska-Gołacka, Sylwia Sowa, Rafał Brudnias, Daniel Paćko, Zbigniew Słomka, Artur Piasek, Piotr Wieciński, Witold Habrat, Halina Garbacz, Agnieszka Kopia, Jerzy Smolik

**Affiliations:** 1 Łukasiewicz Research Network—Institute for Sustainable Technologies, ul. Pułaskiego 6/10, 26-600 Radom, Poland; sylwia.sowa@itee.lukasiewicz.gov.pl (S.S.); rafal.brudnias@itee.lukasiewicz.gov.pl (R.B.); daniel.packo@itee.lukasiewicz.gov.pl (D.P.); zbigniew.slomka@itee.lukasiewicz.gov.pl (Z.S.); artur.piasek@itee.lukasiewicz.gov.pl (A.P.); jerzy.smolik@itee.lukasiewicz.gov.pl (J.S.); 2 Faculty of Chemistry, Warsaw University of Technology, ul. Noakowskiego 3, 00-664 Warsaw, Poland; piotr.wiecinski@gmail.com; 3 The Faculty of Mechanical Engineering and Aeronautics, Rzeszów University of Technology, Powstańców Warszawy 12 St., 35-959 Rzeszów, Poland; witekhab@prz.edu.pl; 4 Faculty of Materials Science and Engineering, Warsaw University of Technology, ul. Wołoska 141, 02-507 Warsaw, Poland; halina.garbacz@pw.edu.pl; 5 Faculty of Metals Engineering and Industrial Computer Science, AGH University of Science and Technology, A. Mickiewicza 30 St., 30-059 Krakow, Poland; kopia@agh.edu.pl

**Keywords:** PVD, thin coatings, anti-wear coatings, cutting tools, thermal conductivity of PVD coating, BUE and BUL formations, cutting processes

## Abstract

Anti-wear coatings obtained through PVD methods may significantly increase the durability of cutting tools by impacting their wear mechanisms. This study presents and discusses the results of studies on the impact of the thermal conductivity of PVD coatings on the intensity of the built-up edge (BUE) and built-up layer (BUL) formation in Inconel 600 alloy machining processes. The authors determine the microstructure, phase structure, mechanical properties (hardness, Young’s modulus, and adhesion), and thermal conductivity of different PVD coatings selected for the purpose of the study and varying in terms of conductivity—i.e., AlCrTiN and AlCrTiN/BN. Machining processes were carried out under controlled conditions using VBGT160404-M3 cutting inserts with AlCrTiN and AlCrTiN/BN coatings deposited on their surface. The authors prove that the adjustment of the thermal conductivity of PVD coatings to the thermal conductivity of the tool and machined materials can help change the direction of heat flow to cool the cutting zone more effectively. The study results presented in this article show that the deposition of the AlCrTiN/BN coating reduces the friction wear on the tool flank by over 70% and lowers the intensity of BUE and BUL formation processes on the face by 10%, compared to the AlCrTiN coating.

## 1. Introduction

Inconel alloys are heat-resistant superalloys (HRSAs) with outstanding physical properties that can withstand elevated temperatures. The most commonly used are nickel, iron, or cobalt-based Inconel alloys, which, nowadays, account for over 50% of the weight of aircraft turbojet engines—a value which is likely to grow in the future, given the current trend.

On the other hand, Inconel alloys are characterized by poor machinability and high instability during machining, which hampers their industrial application [1]. This is mainly caused by their high chemical affinity to steel, work hardening as a result of plastic deformation, and low thermal conductivity. However, it should be remembered that this is not true for all Inconel alloys, as they differ in terms of their chemical composition, microstructure, and mechanical properties.

High chemical affinity to steel means that Inconel alloys may bond with and build up on the working surface of tools [2]. Work hardening as a result of plastic deformation and low thermal conductivity make the machining of Inconel alloys difficult. Additionally, the presence of hard carbide phases in the Inconel alloy microstructure significantly increases tool blade wear at the time of machining. Low thermal conductivity hampers heat transfer from the cutting zone, which impacts the condition of both the machined material as well as the material of which the tool is made. High tensile strength precludes deep cutting [3,4], while the above-mentioned work hardening as a result of plastic deformation requires cutting at a depth greater than the thickness of the work-hardening zone. Studies in this area [5] confirmed that nickel-based alloys work harden during machining, which is caused by a significant plastic deformation of the front of the blade. The size of the deformation depends on the cutting speed and it varies as a function of distance from the machined surface. 

The general description of the tool wear mechanism at the time of Inconel alloy cutting shows that, at a low cutting speed, the friction wear mechanism is dominant [6,7,8]. As the cutting speed and feed value increase, the temperature at the tool-machined material interface significantly increases [9,10,11]. Diffusion processes are growing in importance. As a result, more favorable conditions enabling the transfer of Ni, Nb, Cr, and Fe from the machined material to the tool surface, adhesion to the cutting edge, flank, and face, and formation of BUEs and BULs are created. BUEs and BULs are not stable, and, frequently, parts of the machined material are removed together with the elements of the tool material, which causes crater wear, chipping, tipping, and fracturing [12,13]. As a result, build-ups are created and torn away—often together with the parts of the tool material. This leads to cyclic strain differences in the tool material and to microcrack propagation and dispersion in the tool. Additionally, this may also result in “pushing the tool out of its original track” and worsen the roughness of the machined surface [14]. As a result of delamination, build-ups and parts of the tool material may penetrate the cutting zone, significantly intensifying friction wear processes and increasing the friction coefficient and cutting forces. All these processes, i.e., adhesive wear, higher roughness, fracture, and increased friction coefficient, cause an even greater increase in temperature in the cutting zone. This in turn allows plastic deformation of the surface layer of the tool material due to its reduced stiffness caused by an elevated temperature. Given the above factors, a temperature increase in the cutting zone, which decides on the intensity of the BUE and BUL formation process, is the main factor affecting the intensity of the cutting tool wear. The comparison of the thermal conductivity of Inconel alloys with a varying chemical composition [15,16] (11.4–14.8 W/m·K) with a thermal conductivity of a WC-Co sintered carbide with different granulations [17] (45–80 W/m·K) indicates a more intensive heat flow from the cutting zone to the tool rather than to the machined material. 

To increase the durability of cutting tools used in the machining of Inconel alloys, hard anti-wear coatings, e.g., multicomponent Al-Ti-N-based PVD coatings, are deposited on their surface [18,19,20]. Coatings containing multi-metal TiAlN nitrides, e.g., TiN/TiAlN)_multilayer_ [21], AlCrTiN, and TiAlSiN [22] coatings, have a thermal conductivity between 3.5 and 4.5 W/m·K. Research results [23,24,25,26,27,28,29,30] and market insights by tech companies [31,32,33,34,35,36] providing PVD services show a growing importance of multicomponent multilayer coatings in increasing the durability of cutting tools used in Inconel alloy machining, including: TiAlSiN, TiAlCrN/TiCrAl_52_Si_8_N, TiAlN/γ-Al_2_O_3_, TiAlCrSiYN/TiAlCrN, TiAlN/VN + 2%Y, *nc*-(Ti,Al)N/*a*-Si_3_N_4_, AlCrSiN/NbN, and AlTiCrSiYN/AlTiCrN. Potential material solutions are suggested with the consideration of the above-presented PVD wear mechanism in the Inconel alloy machining process, and most frequently they constitute a modification of AlTiN coating. A properly designed PVD coating also decreases the friction coefficient and ensures a lower chemical affinity to the machined material than the tool material, which makes the transfer of chemical elements from the machined material to the tool surface less intensive, increasing the stability of the rolling force over time. However, it must be remembered that coating materials with TiAlN have a significantly lower thermal conductivity coefficient (3.5–4.5 W/m·K) than sintered carbide (45–80 W/m·K). Given the low thermal conductivity of the machined material—Inconel alloy (11.4–14.8 W/m·K)—this will slow the heat transfer even more and significantly increase the temperature in the cutting zone. 

**Figure 1:**
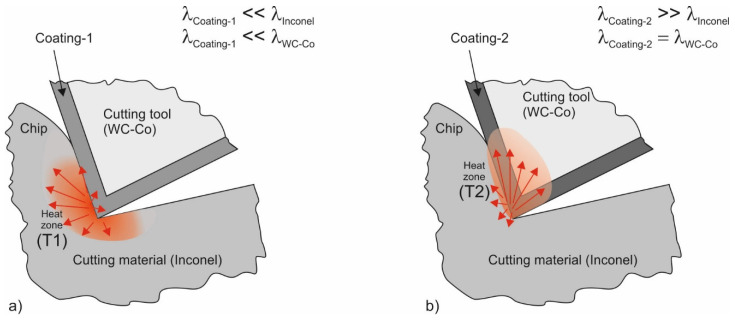
Schematic representation of the intensity and direction of heat transfer from the cutting zone, depending on the thermal conductivity of the PVD coating: (**a**) λ_COATING-1_ << λ_INCONEL_ and λ_COATING-1_ << λ_WC/Co_; (**b**) λ_COATING-2_ >> λ_INCONEL_ and λ_COATING-2_ ≈ λ_WC/Co_.

The production of a hard anti-wear coating, which has high resistance to friction wear and, at the same time, the highest thermal conductivity possible, seems to be the best solution to increase the durability of cutting tools used for the purpose of Inconel alloy machining. According to the assumption shown schematically in Figure 1, PVD coatings with varying thermal conductivities will cause heat to transfer from the cutting zone at different speeds and in different directions. 

In the case of low-thermal-conductivity Coating-1 (Figure 1a), where λ_COATING-1_ << λ_INCONEL_ and λ_COATING-1_ << λ_WC/Co_, the coating will constitute a barrier hampering the heat flow between the cutting zone and the tool, which will result in heat accumulation in the cutting zone. In the case of high-thermal-conductivity Coating-2 (Figure 1b), where λ_COATING-2_ >> λ_INCONEL_ and λ_COATING-2_ ≈ λ_WC/Co_, heat will be transported to the tool effectively. The temperature in the friction zone between the tool and coating characterized by lower thermal conductivity will be significantly higher: T1 >> T2. The studies presented in this article aimed to analyze the impact of an anti-wear coating with increased thermal conductivity on the intensity of the built-up edge (BUE) and built-up layer (BUL) formation in selected Inconel alloy friction and machining processes. The obtained results contribute to the development of material solutions that improve the durability of cutting tools used in the Inconel machining process.

## 2. Materials and Methods

### 2.1. Coating Materials

**Figure 2:**
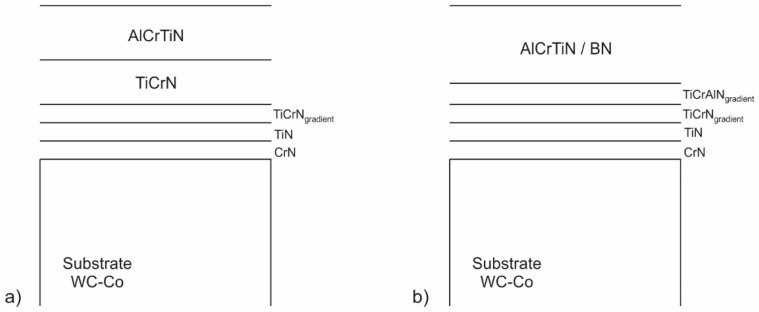
Diagram of the structure of coatings selected for the purpose of the study: (**a**) AlCrTiN; (**b**) AlCrTiN/BN.

For the purpose of the study, two PVD coatings were selected, i.e., AlCrTiN and AlCrTiN/BN. The coatings had a multilayer structure (Figure 2a,b).

**Figure 3:**
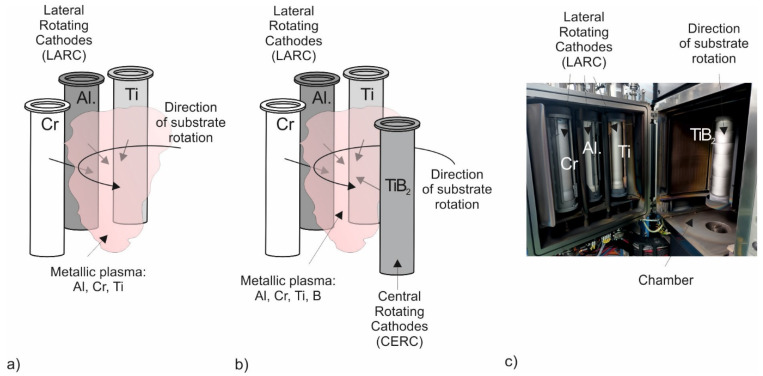
Chemical composition of plasma source cathodes and their distribution in the process chamber at the time of production of selected PVD coatings: (**a**) AlCrTiN and (**b**) AlCrTiN/BN; (**c**) real view of plasma sources in the Pi 411 Plus coating unit chamber.

All coatings studied were produced on a Pi411 Plus coating unit by PLATIT. The AlCrTiN coating was obtained through Arc Evaporation using lateral rotating cathodes (LARCs), and the AlCrTiN/BN coating was obtained through Arc Evaporation + Magnetron Sputtering using LARCs and sputtered coating induced by lateral glow (SCiL) with a TiB_2_ cathode. The chemical composition of the cathodes of individual plasma sources and their distribution in the process chamber at the time of selected PVD-coating production are presented in Figure 3a–c; process parameters are shown in Table 1.

Coatings selected for the purpose of the study were made on three types of samples:

- Samples made of WC-Co (VHM) carbide with a diameter of ϕ = 25 mm, thickness of g = 6 mm, and surface roughness of Ra = 0.05 μm, intended for the analysis of the mechanical properties, chemical composition, and microstructure of coatings;
- Monocrystalline Si (100) samples (size: 10 × 10 mm; thickness: g = 0.1 mm) intended for the analysis of the thermal conductivity coefficient; and
- VBGT160404-M3 cutting inserts by SECO intended for cutting tests.

### 2.2. Material Investigations

#### 2.2.1. Coating Thickness Measurement

The thickness of the obtained PVD coatings was assessed using the ball cratering method, which involves grinding a small crater in a coating with a ball, deeper than the coating thickness, and observing it under an optical microscope to measure the diameter of individual coating boundaries and determine the coating thickness using Equation (1). To assess the thickness of the analyzed PVD coatings, craters were made using a CALOWER ball crater device by CSM Instruments and a ball with a diameter of 25 mm.

(1) $$g_{c}={\left[(d_{1}\right)}^{2}-{\left(d_{2}\right)]}^{2}/8\cdot R$$

*g_c_*—coating thickness;

*d*_1_—diameter of the crater on the surface of the coating;

*d*_2_—diameter of the crater at the coating–substrate interface;

*R*—ball radius.

#### 2.2.2. Hardness and Young’s Modulus

The hardness and Young’s modulus of the coatings were measured using an Anton Parr NHT nanoindentation tester and a Berkovich intender. To eliminate the impact of the substrate parameters on the measurement results, the tests were conducted for limited indentation (less than 10% of the total coating thickness). A total of 15 hardness and Young’s modulus tests were carried out for each test sample. Then, mean values were determined for the 10 representative measurements. H/E and H^3^/E^2^ ratios were calculated. The H/E ratio determines the elastic strain of the coating, while the H^3^/E^2^ ratio represents its resistance to plastic deformation; the increase in the value of these ratios improves in the load bearing capacity.

#### 2.2.3. Adhesion

Coating adhesion was determined using an Anton Parr REVETEST scratch tester (Anton Parr, Tokyo, Japan) with a Rockwell penetrator. Scratches were made under linear penetrator load increase between 0 and 200 N and constant load increase speed of ΔF = 10 N/mm. Values of critical forces [37]: Fn_1_—indenter load at which the internal cohesion of the coating is lost and the first cracks appear; Fn_2_—indenter load at which the coating loses its adhesion to the substrate and the first defects appear; and Fn_3_—indenter load at which the coating is completely removed from the scratch zone—determined based on the analysis of changes in the friction coefficient and acoustic emission (AE), as well as the observation of scratches under a HITACHI TM 3000 scanning microscope (Hitachi High-Tech, Tokyo, Japan). 

#### 2.2.4. Roughness

Surface roughness and topography tests were carried out for all PVD coatings produced. To this end, a KEYENCE VHX1000 digital microscope (Keyence, Osaka, Japan) was used.

#### 2.2.5. Thermal Conductivity

Thermal conductivity tests were conducted in two phases. First, the authors determined the thermal diffusivity of the coating materials on a Laser Flash Apparatus LFA-427 by NETZSCH-Gerätebau GmbH (Selb, Germany). The laser flash method measures the heat on the rear side of the sample absorbed after the front side of the sample is heated by an energy pulse. For the purpose of the study, the authors used a pulsed Nd:YAG laser with a wavelength of 1064 nm and energy of up to 20 J/imp. The LFA-427 apparatus used allows the measurement of thermal diffusivity in the range from 0.001 to 10 cm^2^/s with an accuracy < 3% at a temperature ranging from room temperature to 1500 °C. The tests were conducted at 25–700 °C on monocrystalline Si samples with the following dimensions: 10 mm × 10 mm × 1 mm. Diffusivity was determined using Equation (2):

(2) $$D=0.1388\cdot \frac{g_{s}}{t_{0.5}}$$

*D*—thermal diffusivity;

*g_s_*—substrate thickness; 

*t*_0.5_—time after which the temperature on the rear side of the sample reaches half of the maximum value.

Based on the determined changes in thermal diffusivity as a function of temperature, and considering the phase structure of the coatings as well as the density and specific heat of each phase, the authors then calculated the thermal conductivity of AlCrTiN and AlCrTiN/BN coatings using Equation (3).

λ = D·S_h_·ρ (3)

λ—thermal conductivity;

D—thermal diffusivity;

S_h_—specific heat;

ρ—density.

#### 2.2.6. Chemical Composition

Chemical composition tests of the multilayer coatings produced were conducted using glow discharge optical emission spectroscopy (GDOES) on a Jobin Yvon Horiba GD Profiler HR. The authors determined the profiles of chemical composition changes as a function of coating deposition time in the direction from the surface to the substrate. 

#### 2.2.7. Phase Composition

Phase composition tests were carried out on an Empyrean DY1061 diffractometer (Malvern Panalytical, Malvern, UK) equipped with a Cu lamp with an X-ray wavelength of λ_Cu_ = 1.54 Å, at a fixed angle of incidence, with the following parameters: 2 theta = 20°–100°, step = 0.03°, t = 12 s, and a = 3°.

#### 2.2.8. Microstructure

Microstructure was analyzed using an FE-SEM Hitachi S5500 microscope (Hitachi High-Tech, Tokyo, Japan). Observations were carried out in the STEM mode—Scanning Transmission Electron Microscopy. Transmission preparations, approx. 100 nm thick, were prepared on a Hitachi NB500 dual-beam scanning microscope.

### 2.3. Cutting Test

For the purpose of the study, the Inconel 600 alloy in an annealed condition was selected. The chromium content was high (14–17%). The Inconel 600 alloy is a standard engineering material for chemical, heat-treating, plastic, and aeronautical industries. A high nickel content (>70%) ensures resistance at high temperatures (up to 1050 °C). The chemical composition of the Inconel 600 alloy is presented in Table 2. Mechanical properties of Inconel 600: R_m_ = 550–750 MPa, A_min_ = 30%, E = 206 GPa, and thermal conductivity: 14.8 W·m^−1^·K^−1^.

**Figure 4:**
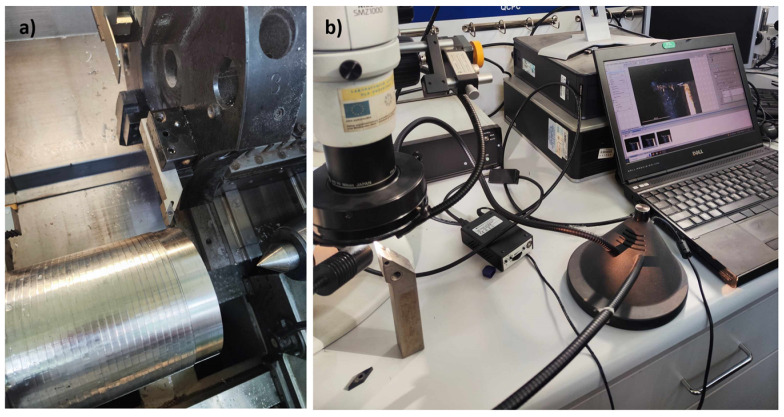
Test stand: (**a**) CNC NEF 600 turning machine; (**b**) tool wear measurement.

Cutting tests were performed on a universal lathe CNC Gildemeister NEF 600 (Maszyneria, Jelenia Góra, Poland) with a Fanuc 200is control system (Figure 4a). Blade wear indices on the VBBmax face were measured using a Nikon SMZ 1000 stereoscopic microscope (Nikon Instruments Inc., Tokyo, Japan) with NIS-Elements AR software (Figure 4b); the analysis of the flank was performed using an Alicona InfiniteFocus optical measurement system (Alicona, Itasca, IL, USA) employing focal differentiation microscopy.

A VBGT160404-M3 cutting insert made of uncoated sintered carbide (HX) was selected for the purpose of the study and coated with the analyzed coatings. The studies were of comparative nature. The cutting insert was mounted using an SVJBL2525M16 tool holder. In accordance with the manufacturer’s recommendations (SECO), the following cutting parameters were adopted: cutting speed—*V_c_* = 20 m/min, feed—*f* = 0.1 mm/rev, and depth of cut—*a_p_* = 0.25 mm.

As a coolant, a semisynthetic Ecocool Global 10 recommended for the machining of metal alloys that are difficult to machine was used. In addition to its very good technological properties, this type of coolant also has good anti-corrosion performance, high stability, and insensitivity to water hardness. As recommended by the supplier, the authors used a coolant concentration between 7% and 9%. The cutting zone was cooled using a flood cooling method.

## 3. Results and Discussion

### 3.1. Mechanical Properties: Thickness, Hardness, Young’s Modulus, Adhesion, and Roughness

**Figure 5:**
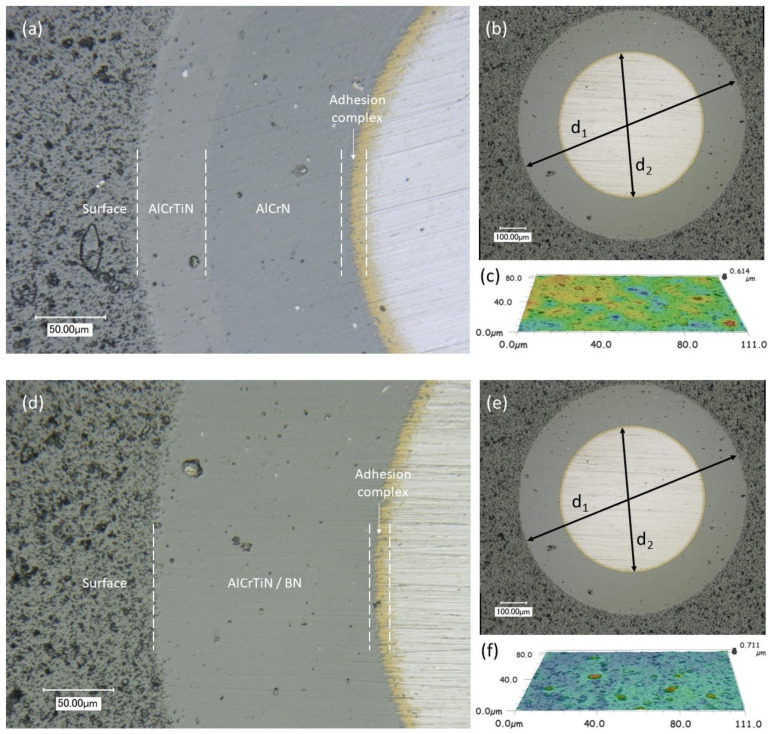
Ball cratering and roughness test results: (**a**–**c**) AlCrTiN; (**d**–**f**) AlCrTiN/BN.

The results of crater observations employing a ball cratering method carried out to initially assess the structure of the produced coatings and measure their thickness are presented in Figure 5a–f.

The thickness of the produced PVD coatings was in the range between g_AlCrTiN_ ≈ 4.30 and g_AlCrTiN/BN_ ≈ 5.35 µm. An analysis of the surface roughness showed the presence of a droplet phase—characteristic spherical irregularities on the surface of the coatings. The topography of the surface of the analyzed coatings is typical of coatings produced by arc evaporation.

The authors indicated that the produced coatings have a hardness of H ≈ 23.6–26.4 GPa and Young’s modulus of E ≈ 390–477 GPa. Varying hardness and Young’s modulus values affect the elastic and plastic properties of the produced coatings, including elastic strain (H/E) and resistance to plastic deformation (H^3^/E^2^). The analyses show that the AlCrTiN/BN coating, for which the H/E and H^3^/E^2^ ratios are higher than in the case of the AlCrTiN coating, has better elastic and plastic properties. This means that the AlCrTiN/BN coating is more resistant to external loads than the AlCrTiN coating.

**Figure 6:**
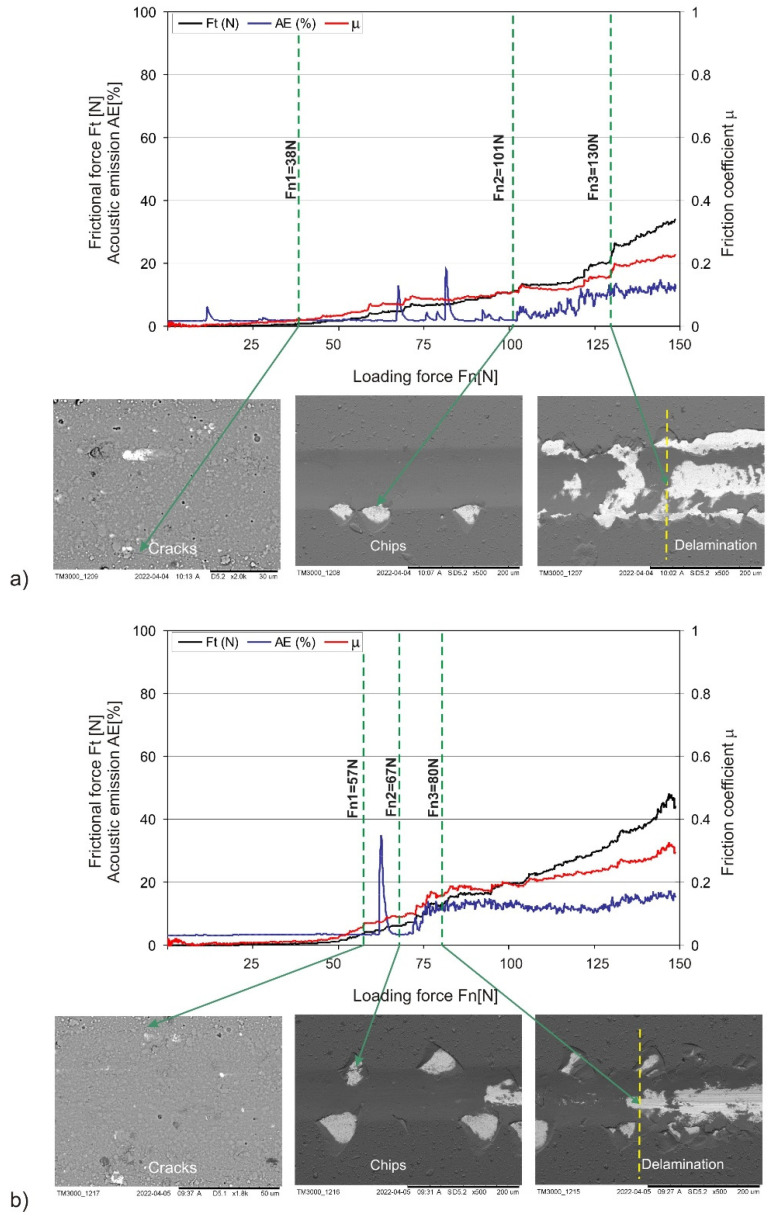
Adhesion test results for PVD coatings: (**a**) AlCrTiN; (**b**) AlCrTiN/BN.

Due to its high resistance to external loads connected with the H/E and H^3^/E^2^ ratios, the AlCrTiN/BN coating exhibits higher resistance to dry friction, which is confirmed by the value of the Fn1 parameter obtained during adhesion scratch tests. It should be assumed that the AlCrTi/BN coating will have greater resistance to cyclically varying loads that cause crack propagation. However, the adhesion of the AlCrTiN/BN coating measured with parameter Fn2 representing the indenter load at which the coating locally loses its adhesion to the substrate was as much as 50% lower than in the case of the AlCrTiN coating (Fn2_TiCrTiN_ = 101 N, Fn2_TiCrTiN/BN_ = 67 N). Higher external-load values may accelerate the delamination of the AlCrTiN/BN coating and expose the substrate. The results of the adhesion tests for AlCrTiN and AlCrTiN/BN coatings are presented in Figure 6a,b.

Mechanical properties of the studied PVD coatings, i.e., thickness, hardness, Young’s modulus, roughness, and adhesion, are presented in Table 3.

### 3.2. Microstructure and Chemical Composition

**Figure 7:**
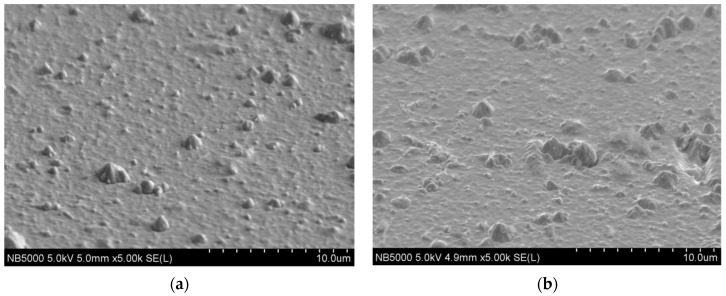
Results of surface observations: (**a**) AlCrTiN; (**b**) AlCrTiN/BN.

SEM images of the surface of the studied AlCrTiN and AlCrTiN/BN coatings are presented in Figure 7a,b. On the surface of both coatings, microdroplets of metals used in the deposition process (Al, Cr, Ti), which form during arc evaporation (AE), are visible. Their size varies between 0.5 and 5 μm; however, most of them are not bigger than 2 μm. No other defects (e.g., cracks or coating delamination) were observed. In the areas between the microdroplets, the coatings have a homogeneous structure and comparable roughness. The morphology of the surface of the analyzed coatings is typical of coatings produced by arc evaporation.

**Figure 8:**
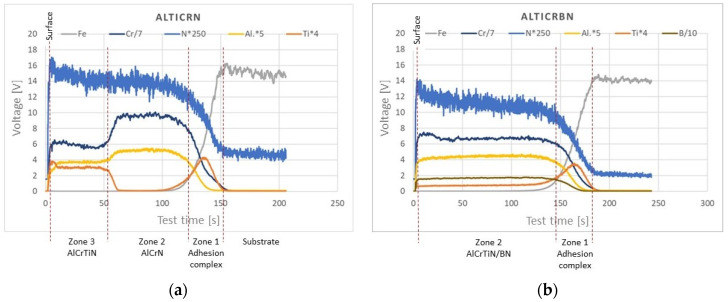
Results of chemical composition analysis employing GDOES: (**a**) AlCrTiN; (**b**) AlCrTiN/BN.

Qualitative analysis of the chemical composition of the AlCrTiN and AlCrTiN/BN coatings carried out by means of GDOES enabled the determination of profiles of changes in chemical composition as a function of distance from the surface. The analysis results are presented in Figure 8a,b. 

Based on the determined profiles of chemical composition changes, it should be concluded that the AlCrTiN coating (Figure 8a) has a three-zone structure. In direct proximity to the substrate, there is zone 1—the adhesion complex. It has a variable content of individual chemical elements and its thickness is below 10% of the coating thickness. The next zone is zone 2—AlCrN—with a thickness ≈ 50% of the coating thickness. Directly under the surface, there is zone 3—AlCrTiN. The AlCrTiN/BN coating (Figure 8b) has a two-zone structure. Analogous to the AlCrTiN coating, in the AlCrTiN/BN coating, zone 1 is the adhesion complex, which also has a variable content of individual chemical elements. Zone 2, i.e., the mixture of Al, Cr, Ti, B, and N, constitutes the remaining 90% of the coating thickness.

**Figure 9:**
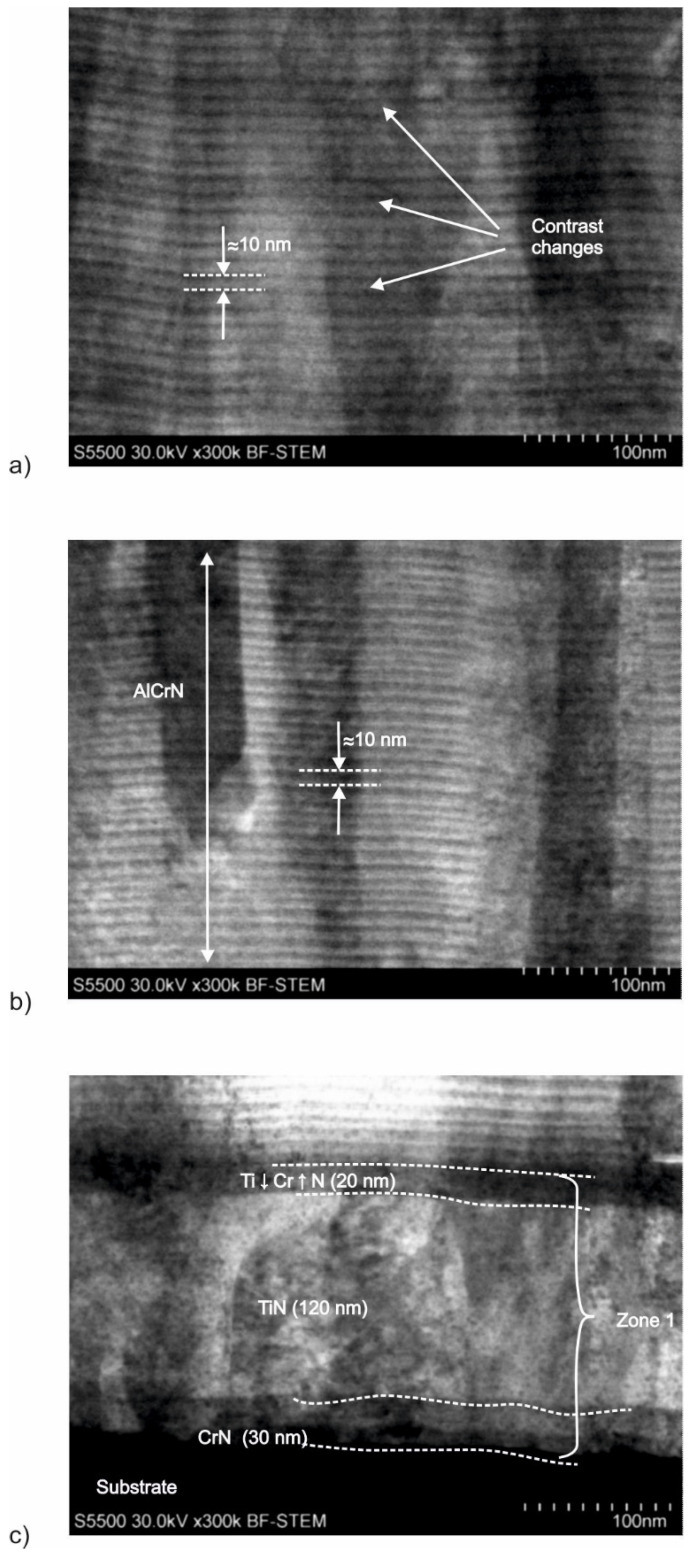
Results of microstructure analysis of the AlCrTiN coating: (**a**) zone 3—AlCrTiN, (**b**) zone 2—AlCrN, and (**c**) zone 1—adhesion complex.

**Figure 10:**
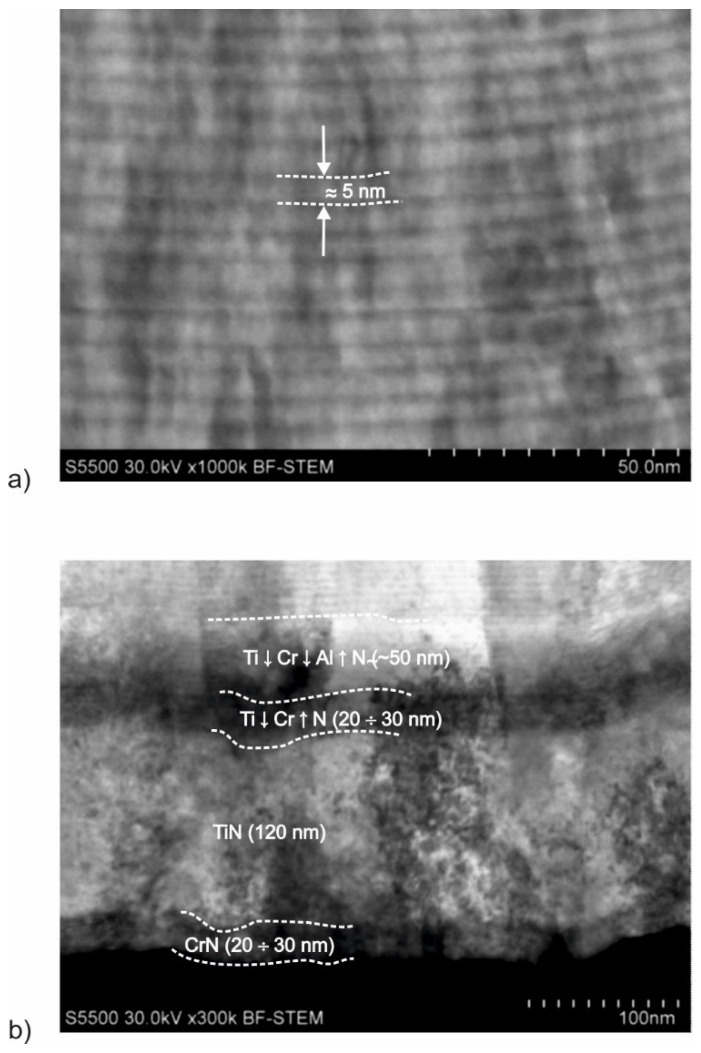
Results of microstructure analysis of the AlCrTiN/BN coating: (**a**) zone 2—AlCrTiBN; (**b**) zone 1—adhesion complex.

Considering the changes in the chemical composition of the AlCrTiN and AlCrTiN/BN coatings (Figure 8) and the process parameters presented in Table 1, the authors determined the characteristics of the structure of individual zones in each coating. The results of the FIB analyses of the microstructures of the AlCrTiN and AlCrTiN/BN-coating cross-sections are presented in Figure 9 and Figure 10, respectively.

The results of the AlCrTiN coating microstructure analysis (Figure 9) show that zone 1, the adhesion complex, located directly adjacent to the substrate, is composed of three layers: CrN (30 nm), TiN (120 nm), and Ti↓Cr↑N_gradient_ (Figure 9c). In regard to the Ti↓Cr↑N_gradient_ layer, it is clearly visible that, in the direction of the coating growth, the titanium content decreases, while the chromium content increases. Zone 2, AlCrN (Figure 9b), which is 1.5 µm thick and located in direct proximity to the adhesion complex, also has a multilayer structure, and each of its layers (AlN and CrN) is ≈10 nm thick. Directly adjacent to the surface, there is zone 3—AlCrTiN (Figure 9a). It is about 2.6–2.7 micrometers thick and has a multilayer structure as well (each of its layers is also approx. 10 nm thick). 

The results of the AlCrTiN/BN-coating microstructure analysis (Figure 10) show that zone 1—the adhesion complex located directly adjacent to the substrate—is composed of four layers (Figure 10b): CrN (30 nm), TiN (120 nm), Ti↓Cr↑N_gradient_ (20–30 nm), and Ti↓Cr↓Al↑N_gradient_ (50 nm). The remaining part of the coating with a thickness between 5.0 and 5.1 µm is made up of zone 2. It is also multilayered and each of its layers is approx. 5 nm thick. The analysis of the chemical composition of the AlCrTiN/BN coating (Figure 8b) shows that zone 2 is a mixture of Al, Cr, Ti, B, and N. Therefore, the visible layers are alternating layers of single-metallic or multi-metallic nitrides with Al, Cr, Ti, and B. The analysis of the microstructure of the PVD coatings confirmed their very complex multilayer structure. The multi-zone structure of the produced coatings is the result of changing process parameters. On the other hand, the multilayer structure of each zone is the result of the movement of the coated substrates relative to plasma sources.

### 3.3. Phase Composition

To ensure the correct analysis of the X-ray test results, the exact depth of X-ray penetration and structure of the studied coatings had to be known. The authors assumed that the depth of X-ray penetration would be comparable to the depth of penetration into the chromium nitride coating. Using Formula (4), the authors determined the depth of X-ray penetration into the chromium nitride (CrN) coating: *Z_CrN_* = 0.60 µm.

(4) $$z_{CrN}=\frac{-ln(1-Gx)}{\mu (\frac{1}{sin(\alpha )}+\frac{1}{sin(2\theta -\alpha )})}$$

where:

*Z*—the depth of X-ray penetration;

*G*—Gaussian module for *CrN* = 0.9;

µ—linear absorption coefficient of *CrN* = 1862.22 [1/cm], α = 3°, 2θ = 43.83°.

**Figure 11:**
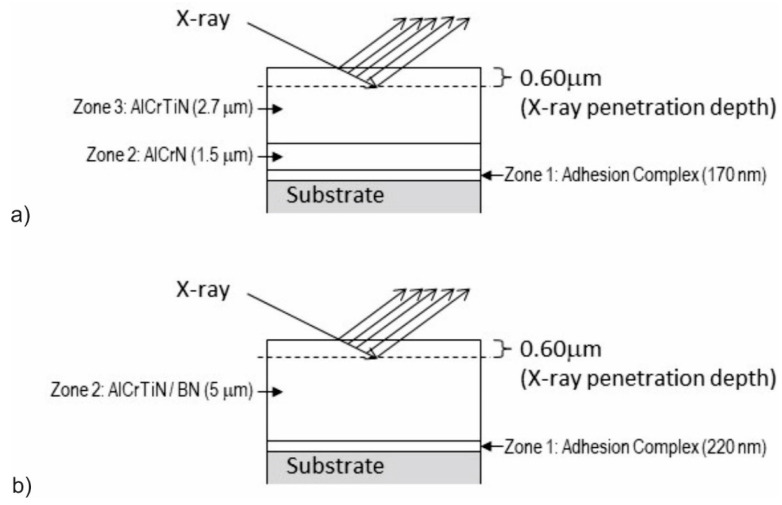
X-ray penetration-zone analysis during phase tests of the produced coatings: (**a**) AlCrTiN; (**b**) AlCrTiBN.

**Figure 12:**
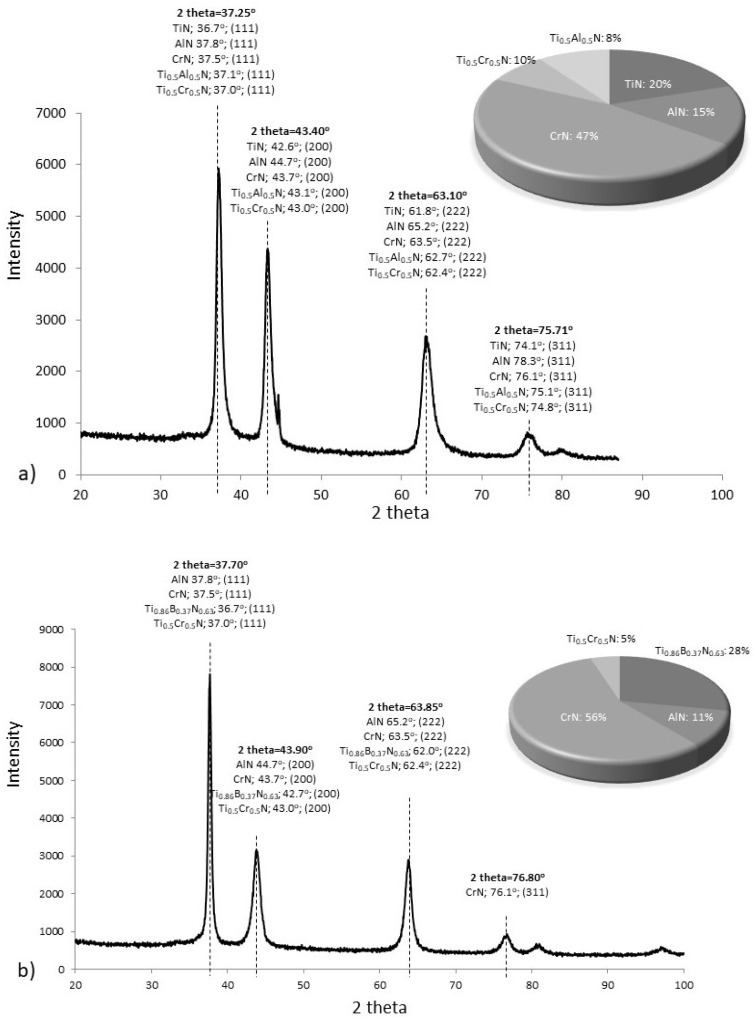
Results of phase structure analyses: (**a**) AlCrTiN coating; (**b**) AlCrTiN/BN coating.

Taking into account the results of the microstructure analyses, including the thickness of individual zones and chemical composition analyses, the authors determined during phase tests the X-ray penetration zone (Figure 11a,b). The results of the phase analysis of the AlCrTiN and AlCrTiN/BN coatings are shown in Figure 12a,b, respectively.

In the case of the AlCrTiN coating (Figure 12a), distinct peaks were recorded for various metal nitrides, including single-metal nitrides TiN, CrN, and AlN, as well as multi-metal nitrides Ti_0.50_Cr_0.50_N and Ti_0.50_Al_0.50_N. The use of HighScore Plus software enabled the authors to determine the content of individual phases, taking into account the field under the peak: TiN: 20%, CrN: 47%, AlN: 15%, Ti_0.50_Cr_0.50_N: 10%, and Ti_0.50_Al_0.50_N: 8%. At the same time, taking into account the complexity of the phase structure and microstructure fragmentation in zone 3 of the AlCrTiN coating (Figure 9a) as well as the results of the studies conducted by Hasegawa et al. [38], the authors identified the recorded peaks as the Ti_x_Cr_y_Al_z_N phase, where: *x* + *y* + *z* = 1.

As for the AlCrTiN/BN coating (Figure 12b), distinct peaks were recorded for single-metal nitrides CrN and AlN, as well as multi-metal nitrides Ti_0.50_Cr_0.50_N and Ti_0.86_B_0.37_N_0.63_. The content of the identified phases was estimated at the following levels: CrN: 56%, AlN: 11%, Ti_0.50_Cr_0.50_N: 5%, and Ti_0.86_B_0.37_N_0.63_: 28%. The diffraction peaks recorded have a similar angular position of 2 theta, as in the diffractogram recorded for the AlCrTiN coating. Taking into account the results of the EDS chemical composition analysis, which showed the presence of boron in the zone adjacent to the surface, the recorded peaks can be interpreted as multi-metallic nitrides with boron, i.e., Ti_x_Cr_y_Al_z_B_t_N, where: *x* + *y* + *z* + *t* = 1.

### 3.4. Thermal Conductivity

Based on the diffusivity test results, taking into account the density (ρ) and specific heat (S_h_) of the coating materials, the value of thermal conductivity (λ) of the produced PVD coatings at temperatures ranging from 25 °C to 700 °C was determined. Density and specific heat of the coating materials were calculated with the consideration of the percentage share of each phase determined in the phase structure analysis (Figure 12a,b). The results of density and specific heat tests are presented in Table 4.

**Figure 13:**
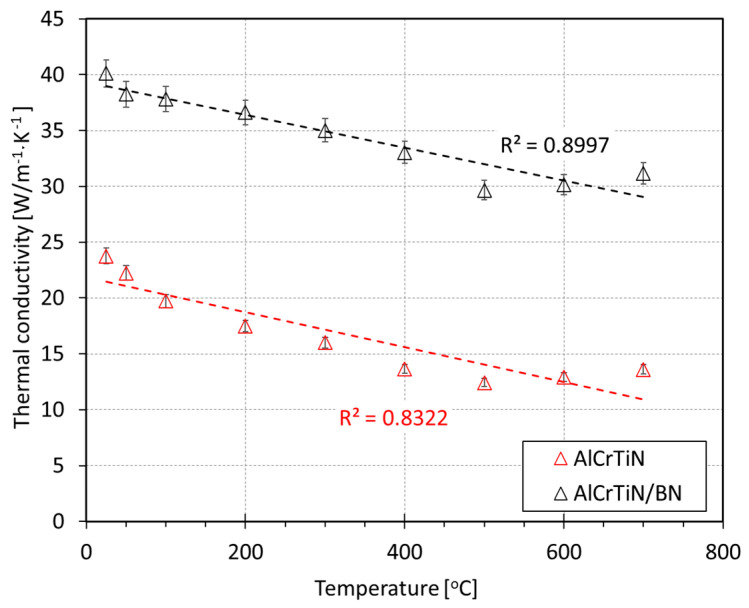
Changes in thermal conductivity of the studied PVD coatingsAlCrTiN and AlCrTiN/BN, as a function of temperature changes (20 °C–700 °C).

Then, the authors determined the value of thermal conductivity of the AlCrTiN and AlCrTiN/BN coatings using Formula (3). For the AlCrTiN coating, thermal conductivity decreases in the entire temperature range studied as the temperature increases, amounting to λ_AlCrTiN(25)_ = 23.76 W·m^−1^·K^−1^ and λ_AlCrTiN(700)_ = 13.62 W·m^−1^·K^−1^. The introduction of the BN phase into the AlCrTiN coating significantly increased thermal conductivity, which, at temperatures of 25 °C and 700 °C, amounted to λ_AlCrTiN/BN(25)_ = 40.13 W·m^−1^·K^−1^ and λ_AlCrTiN/BN(700)_ = 31.18 W·m^−1^·K^−1^, respectively. Changes in the thermal conductivity of the AlCrTiN and AlCrTiN/BN coatings calculated based on changes in diffusivity are presented in Figure 13.

A more than 2.5-times-higher thermal conductivity of the AlCrTiN/BN coating at 700 °C will ensure better heat transfer from the cutting zone to the tool with WC-Co than the AlCrTiN coating. The increase in the thermal conductivity of the tested coatings when the temperature is higher than 500 °C, observed in Figure 13, was caused by an increase in the radiative heat transport mechanism, which is characteristic of ceramic materials [39,40,41].

### 3.5. Cutting Test

**Figure 14:**
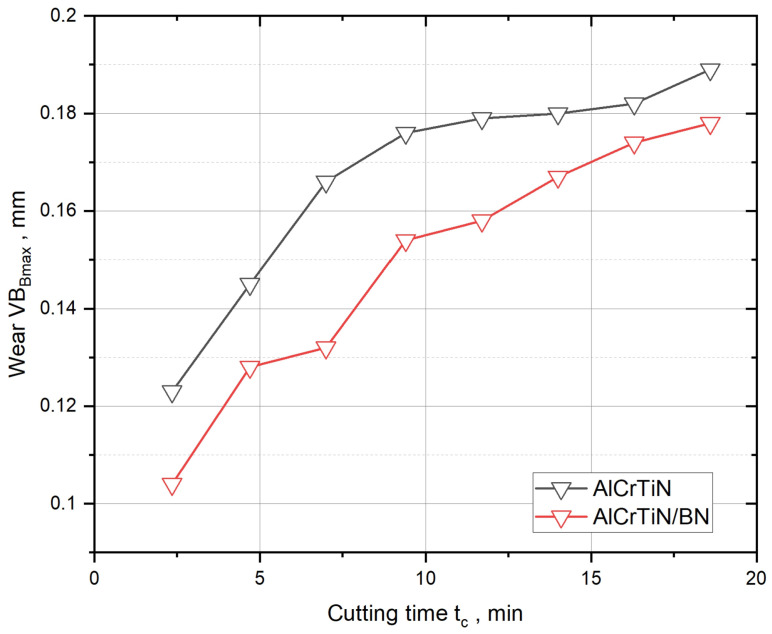
Comparison of changes in the values of the VB_Bmax_ wear index over cutting time for tools with different anti-wear coatings: AlCrTiN and AlCrTiN/BN.

As a part of service tests, the authors specified the dynamics of blade wear, by determining the changes in the VB_Bmax_ value over time on the face. For time of operation, t = 18 min, significantly lower tool wear was observed in the case of the AlCrTiN/BN coating than in the case of the AlCrTiN coating (Figure 14). This is especially evident at the early stages of operation. As the cutting time increases, the differences in VB_Bmax_ wear index values for tools with AlCrTiN and AlCrTiN/BN coatings decrease. These changes may result from the faster removal of the AlCrTiN/BN coating from the tool surface compared to the AlCrTiN coating, which may be due to the lower adhesion of the AlCrTiN/BN coating (Table 3).

**Figure 15:**
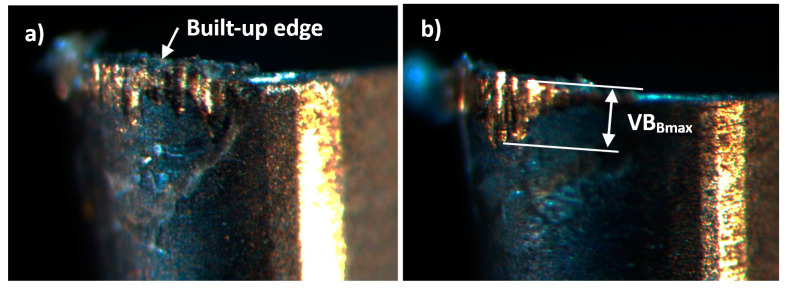
Wear of the flank of tools coated with (**a**) AlCrTiN and (**b**) AlCrTiN/BN after 10 min of operation.

At the same time, the microscopic analysis shows that, in the case of a tool coated with AlCrTiN, scratches on the face are wider and deeper (Figure 15a), which indicates that the dominant wear mechanisms are adhesion or attrition wear. Lower thermal conductivity of the AlCrTiN coating will result in heat accumulation in the cutting zone. At an elevated temperature, as a result of adhesion, spot welds form on the cutting edge at the tool–chip interface. This leads to BUE formation on the cutting edge and flank. The movement of the chip on the BUE causes the weld and some of the tool material to crack and results in the cyclical, local removal of the BUE and part of the coating material. When such a wear mechanism occurs, the tool wears out faster and in a manner that is difficult to predict.

Traces visible on the face of the tool with the AlCrTiN/BN coating indicate the dominance of the abrasion wear mechanism (Figure 15b). Hard material in contact with the cutting tool causes the removal of the tool material through abrasion. This wear is easier to predict and ensures stable hardness of the tool. This confirms the significantly greater resistance of the tool with the AlCrTiN/BN coating to BUE and BUL formation.

**Figure 16:**
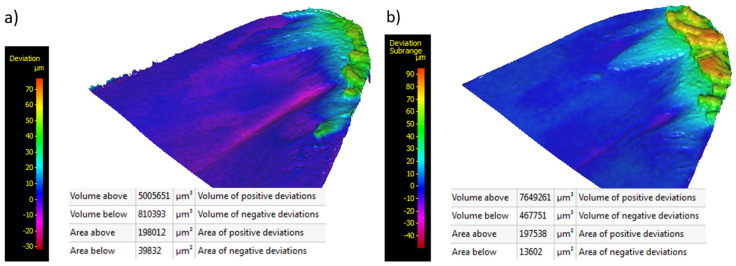
Effect of BUE formation and abrasive wear on the flank of tools with different anti-wear coatings after time t = 18 min: (**a**) AlCrTiN; (**b**) AlCrTiN/BN.

The results of the microscopic analysis of the flank of tools with the AlCrTiN and AlCrTiN/BN coatings (Figure 16a,b) also confirm differences in the BUE formation process. In both cases, BUEs accumulate near the cutting edge and take up a similar area (area above). However, in the case of tools with the AlCrTiN coating (Figure 16a), a BUE on the flank has a volume (volume above) lower by 34% compared to tools with the AlCrTiN/BN coating (Figure 16b). At the same time, AlCrTiN-coated tools are characterized by an over 70% higher volume of tool material loss on the flank (volume below), which occupies an area (area below) three-times larger than in the case of AlCrTiN/BN-coated tools. This confirms the greater dynamics of BUE formation and removal processes in the case of AlCrTiN-coated tools, and lower susceptibility to BUE formation of AlCrTiN/BN-coated tools. Additionally, this also demonstrates the effectiveness of the AlCrTiN/BN coating in the process of increasing the cutting tool life at the time of Inconel 600 alloy machining, until its removal from the tool surface as a result of abrasive wear.

## 4. Conclusions

From the conducted analyses it follows that:

1. AlCrTiN- and AlCrTiN/BN-coated cutting tools have different thermal conductivity values and a tendency to form BUEs and BULs in the Inconel alloy cutting process. Differences in build-up formation mechanisms may be caused by different distributions of heat from the cutting zone.
2. In the Inconel 600 alloy machining process, AlCrTiN-coated tools with a thermal conductivity (λAlCrTiN(700) = 13.62 W·m^−1^·K^−1^) lower than the thermal conductivity of the tool material (λWC-Co = ≈45 W·m^−1^·K^−1^) are more susceptible to BUE formation than tools with the AlCrTiN/BN coating whose thermal conductivity is much higher (λAlCrTiN/BN(700) = 31.18 W·m^−1^·K^−1^) and comparable to the thermal conductivity of the tool material.
3. The improvement of the solution: WC/Co tool–AlCrTiN/BN coating should focus on increasing the coating’s resistance to abrasion wear and its adhesion to the tool material, as a result of which the cutting edge would be coated for a longer period of time. Further temperature decreases in the cutting zone may be achieved by using high-pressure cooling. On the one hand, this will speed up the heat transfer from the cutting zone; on the other hand, it will shorten the tool–chip interface, which should extend the tool life.
4. The experiments carried out allow us to conclude that the modification of the thermal conductivity of anti-wear coatings deposited on the working surfaces of cutting tools enables changing the direction and intensity of heat distribution generated in the cutting zone.

## Figures and Tables

**Table 1:** PVD-coating production process parameters.

Material	Atmosphere	Pressure p [mbar]	U_bias_ [V]	Cathode Current I [A]	Temperature T [°C]	Type of Plasma Source
AlCrTiN coating						
CrN	N_2_:300sccm	8.5 × 10^−3^	−100	Cr:125		LARC(Cr)
TiN	N_2_:350sccm	1.2 × 10^−2^	−60	Ti:150	485	LARC(Ti)
Ti↓Cr↑N_gradient_	N_2_:350sccm	1.2 × 10^−2^	−100	Ti:120↓, Cr:125↑		LARC(Ti,Cr)
TiCrN	N_2_:350sccm	1.2 × 10^−2^	−100	Ti:120, Cr:125		LARC(Ti,Cr)
AlCrTiN	N_2_:500sccm	3.5 × 10^−2^	−45	Al:90, Ti:175, Cr:90		LARC(Al,Cr,Ti)
AlCrTiN/BN coating						
CrN	N_2_:300sccm	8.5 × 10^−3^	−100	Cr:125		LARC(Cr)
TiN	N_2_:350sccm	1.2 × 10^−2^	−60	Ti:150		LARC(Ti)
Ti↓Cr↑N_gradient_	N_2_:350sccm	1.2 × 10^−2^	−100	Ti:120↓, Cr:125↑	480	LARC(Ti,Cr)
Ti↓Cr↓Al↑N_gradient_				Cr:170↓, Al:90↑		LARC(Al,Cr,Ti)
AlCrTiN/BN	N_2_:500sccm	3.5 × 10^−2^	−60	Al:90, Ti:175, Cr:90, TiB_2_:15		LARC(Al,Cr,Ti) SCiL(TiB_2_)

**Table 2:** Chemical composition of Inconel 600.

	Cr	Fe	Mn	Si	Cu	Ti	Al	S	C	Ni
Min.	14	6	-	-	-	-	-	-	-	Bal.
Max.	17	10	1.00	0.50	0.50	0.30	0.30	0.15	0.08	Bal.

**Table 3:** Mechanical properties: thickness, hardness, Young’s modulus, adhesion, and roughness.

Coating	Thickness g [μm]	Roughness [μm]	Hardness H [GPa]	Young’s Modulus E [GPa]	H/E	H^3^/E^2^	Adhesion Fn_1_/Fn_2_/Fn_3_ [N]
AlCrTiN	4.30	0.614	23.6	477	0.049	0.058	38/101/130
AlCrTiN/BN	5.35	0.711	26.4	390	0.068	0.121	57/67/80

**Table 4:** Density and specific heat of the materials of AlCrTiN and AlCrTiN/BN coatings calculated with the consideration of the percentage share of individual phases.

*nc*-AlCrTiN						
Phase composition	TiN	Ti_0.5_Cr_0.5_N	Ti_0.5_Al_0.5_N	AlN	CrN	Average value
Specific heat S_h_ [J/g·°C]	0.604	0.651	0.6675	0.732	0.699	0.69
Density ρ [g/cm^2^]	5.22	5.30	3.85	3.20	6.10	4.66
Percentage	20%	10%	8%	15%	47%	
*nc*-AlCrTiN/BN						
Phase composition	Ti_0.86_B_0.37_N_0.63_	CrN	AlN	Ti_0.5_Cr_0.5_N		Average value
Specific heat S_h_ [J/g·°C]	0.668	0.699	0.732	0.651		0.69
Density ρ [g/cm^2^]	3.52	6.10	3.20	5.30		5.02
Percentage	28%	56%	11%	5%		

## Data Availability

Data are contained within the article.
